# Regulation of microRNA biogenesis and turnover by animals and their viruses

**DOI:** 10.1007/s00018-012-1257-1

**Published:** 2013-01-26

**Authors:** Valentina Libri, Pascal Miesen, Ronald P. van Rij, Amy H. Buck

**Affiliations:** 1 Centre for Immunity, Infection and Evolution, University of Edinburgh, King’s Buildings, West Mains Road, Edinburgh, EH9 3JT, UK; 2 Centre for Human Immunology, Immunology Department, Pasteur Institute, 25 rue du Docteur Roux, 75015 Paris, France; 3 Department of Medical Microbiology, Nijmegen Centre for Molecular Life Sciences, Radboud University Nijmegen Medical Centre, 6500 HB, Nijmegen, The Netherlands

**Keywords:** MicroRNA, MicroRNA biogenesis, MicroRNA turnover, RNA degradation, Herpesviruses, Host–pathogen, Viral suppressor of RNA interference

## Abstract

MicroRNAs (miRNAs) are a ubiquitous component of gene regulatory networks that modulate the precise amounts of proteins expressed in a cell. Despite their small size, miRNA genes contain various recognition elements that enable specificity in when, where and to what extent they are expressed. The importance of precise control of miRNA expression is underscored by functional studies in model organisms and by the association between miRNA mis-expression and disease. In the last decade, identification of the pathways by which miRNAs are produced, matured and turned-over has revealed many aspects of their biogenesis that are subject to regulation. Studies in viral systems have revealed a range of mechanisms by which viruses target these pathways through viral proteins or non-coding RNAs in order to regulate cellular gene expression. In parallel, a field of study has evolved around the activation and suppression of antiviral RNA interference (RNAi) by viruses. Virus encoded suppressors of RNAi can impact miRNA biogenesis in cases where miRNA and small interfering RNA pathways converge. Here we review the literature on the mechanisms by which miRNA biogenesis and turnover are regulated in animals and the diverse strategies that viruses use to subvert or inhibit these processes.

## Introduction

### Small RNA classification

The specific recognition of nucleic acid sequences by RNA–protein complexes (RNPs) is central to transcriptional and post-transcriptional gene regulation. Small RNAs are incorporated into many RNPs in order to mediate the specific recognition of target nucleic acids through Watson–Crick base-pairing. Different classes of small RNAs continue to be discovered, including some that are specific to plants or animal lineages, reviewed in [1, 2]. There are three major classes in animals: microRNAs (miRNAs), short interfering RNAs (siRNAs), and piwi-interacting RNAs (piRNAs). These classes differ in their origin and biogenesis, the proteins with which they interact, the mechanism of action of the RNP in which they are contained, and the nature of their targets. MiRNAs are derived from single-stranded (ss) RNAs that fold back on themselves into stem-loop structures. Endogenous siRNAs originate from double-stranded (ds) RNA precursors that result from convergent bi-directional transcription, inverted repeat regions in structured RNA, or base-pairing between protein-coding genes and pseudogene-derived antisense transcripts. The detailed mechanism(s) of piRNA biogenesis remains somewhat elusive, but the primary piRNAs originate from single-stranded precursor RNAs and are only found in animals, and specifically in the germline [3]. Each class of small RNAs binds to a member of the Argonaute (Ago) family of proteins: siRNAs and miRNAs associate with the Ago clade, whereas piRNAs associate with the Piwi clade, reviewed in [4]. The Ago protein bound to the small RNA comprises the RNA-induced silencing complex (RISC). There is increasing diversity in the mechanisms by which RISCs function and in the genes they target [5]. The RISCs containing miRNAs are found throughout the eukaryal domain and primarily target messenger RNAs (mRNAs), causing the inhibition of translation and/or de-adenylation and degradation of the mRNAs, reviewed in [6]. Recognition of the mRNA target does not require perfect complementarity with the miRNA and is generally dictated by the “seed region” within the 5′ terminal region of the miRNA (nucleotides 2–8), reviewed in [7]. Based on this low sequence requirement for recognition, each miRNA is predicted to target several hundred genes. The majority of human protein-coding genes have miRNA binding sites that are maintained under selective pressure [8].

### miRNAs in hosts and viruses

Based on the large number of genes targeted by miRNAs, together with the ability of miRNAs to operate synergistically with one another, these small RNAs are involved in regulating numerous aspects of cellular biology including proliferation, tumorigenesis, metabolism, differentiation, development, apoptosis, and innate and adaptive immune responses, reviewed in [9–14]. Viruses have evolved to exploit and manipulate these same cellular pathways. Therefore, it is not surprising that they use the miRNA pathway to do this, either by encoding their own miRNAs, or encoding molecules that activate or inhibit cellular miRNA expression. Seven different virus families have been reported to encode miRNAs or miRNA-like molecules: herpesviruses, polyomaviruses, adenoviruses, baculoviruses, an ascovirus, and recently a retrovirus and a flavivirus [15–18]. Analysis of a wide range of RNA viruses failed to identify viral miRNAs [17], apart from the identification of miRNAs in bovine leukemia virus (BLV), a retrovirus that replicates in the nucleus [18] and the identification of a miRNA-like species in West Nile virus, a cytoplasmic RNA virus that encodes a stem-loop structure in its 3′UTR [16]. In the latter study the small RNA was detected in infected mosquito cells, but not infected mammalian cells, raising the question of how biogenesis factors differ in the two animals. There have been several reports, some controversial, suggesting that additional retroviruses may encode miRNAs [19–21], but it remains unclear if this strategy would be advantageous to cytoplasmic RNA viruses [17]. However, both DNA and RNA viruses can modulate the expression of host miRNAs to enhance replication or facilitate the progression through their life cycles, reviewed in [22].

**Fig. 1:**
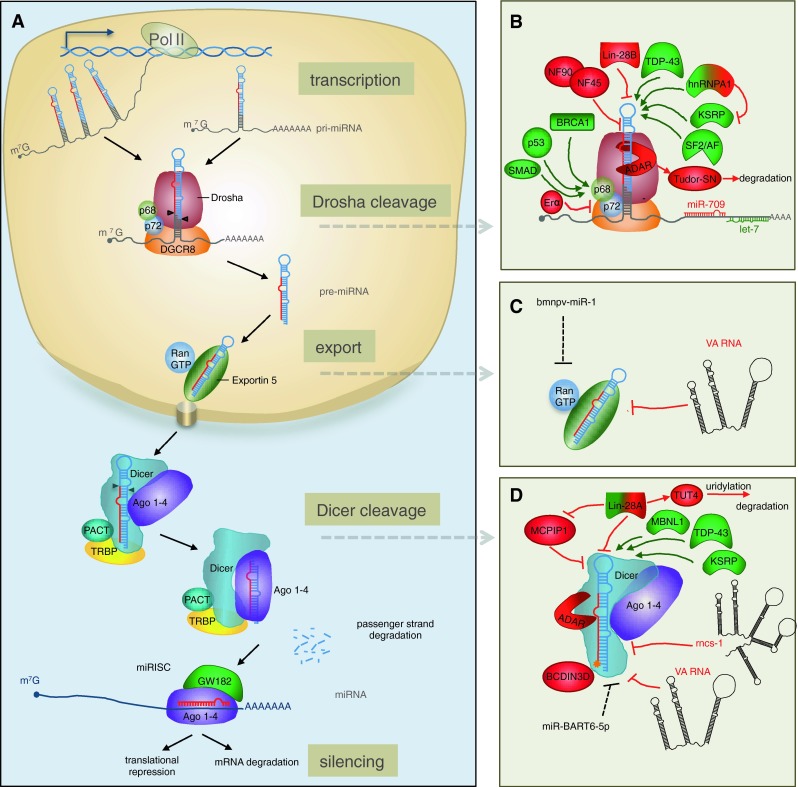
Schematic overview of microRNA biogenesis and regulation in animals. **a** The canonical biogenesis pathway. Pri-miRNAs are transcribed in the nucleus by polymerase II with a cap (m^7^G, 7-methylguanosine-cap) and poly A tail. The pri-miRNA can harbour a single pre-miRNA or a cluster of pre-miRNAs; the mature miRNA sequence is depicted in *red*. Cleavage of the pri-miRNA occurs in the nucleus by the Microprocessor complex, composed minimally of Drosha and DGCR8, which interact with helicases p68 and p72. The pre-miRNA is then exported through the nuclear pore complex into the cytoplasm where the stem is cleaved by Dicer, supported by TRBP or PACT. The miRNA/miRNA* duplex is loaded into the Ago protein within RISC, where one part of the strand is preferentially retained; this complex contains an Ago protein and GW182, which is required for gene silencing. **b** Regulation of pri-miRNA cleavage. Proteins can either positively (*green*) or negatively (*red*) influence cleavage of pri-miRNAs by Drosha, based on direct interactions with the pri-miRNA or interactions with auxiliary proteins p68/p72 (indicated by *arrows*). Factors depicted in both *green* and *red* can behave as positive or negative regulators depending on the identity of the miRNA and the presence of other factors. Mature miRNAs can also regulate pri-miRNA processing through interactions downstream of the stem-loop: let-7 promotes processing of pri-let-7 whereas miR-709 inhibits processing of pri-miR 15/16. **c** Regulation of pre-miRNA export. Two viral non-coding RNAs inhibit miRNA translocation to the cytoplasm: VA1 competes with endogenous pre-miRNAs for binding to Exportin-5 whereas the viral miRNA, Bmnp-miR-1, regulates export indirectly (*dotted line*) by targeting RanGTP. **d** Regulation of pre-miRNA cleavage by Dicer. Proteins that regulate Dicer processing include: (1) Lin28 (Lin28A), which recruits TUT4 that oligo-uridylates pre-miRNAs leading to degradation, (2) MCPIP1 which cleaves the loop, (3) TDP-43 and KSRP, which bind to the loops of both pri-miRNAs and pre-miRNAs and (4) BCDIN3D, which can add methyl groups to the 5′ end of pre-miRNA and inhibit recognition by Dicer. The RNA factors that are known to inhibit Dicer processing include an ~800 non-coding RNA termed rnsc-1, VA RNAs from Vaccinia virus (*black*) and a viral miRNA that regulates Dicer indirectly (*dotted line*)

Given the intricate role of miRNAs in regulating cell biology, it is not surprising that miRNA expression is subject to various levels of regulation, which viruses can also exploit. miRNA biogenesis encompasses a series of sequential processing steps to convert the primary miRNA (pri-miRNA) transcript into the biologically active, mature miRNA (Fig. 1), reviewed in [1, 5]. Following transcription, the pri-miRNA is cleaved by the RNase III-like enzyme Drosha in the nucleus [23] to generate a ~60–70 nt precursor miRNA (pre-miRNA). The pre-miRNA is then exported into the cytoplasm [24] and processed into a ~22 nt duplex by the RNase III-like enzyme Dicer [25–29]. One strand of this duplex is then loaded into RISC which is comprised of at least one Ago protein [30, 31] and GW182, a glycine–tryptophan repeat containing protein required for gene silencing (also known as trinucleotide repeat containing 6, TNRC6) [32]. Each stage in the miRNA biogenesis pathway is subject to regulation. Here we summarise the current literature on the regulation of miRNA biogenesis and turnover and detail the mechanisms by which viruses exploit or manipulate these processes. We focus primarily on animal miRNAs, but highlight some common and distinct properties of plant miRNAs, which evolved separately [33].

## Regulation of miRNA transcription

The first regulatory layer governing miRNA abundance occurs at the stage of transcription of the pri-miRNA. The stem-loop structures from which miRNAs are derived are disseminated throughout the genome, either within intronic sequences of protein-coding genes, within intronic or exonic regions of noncoding RNAs, or set between independent transcription units (intergenic). The majority of intronic miRNAs are transcribed from the same promoter as the host gene. However, approximately one-third of intronic miRNAs are transcribed from independent promoters, enabling separate control of their transcription [34–36]. Most pri-miRNAs are transcribed by RNA polymerase II (Pol II) [37], however, a subset of miRNAs, including viral miRNAs, are transcribed by Pol III [35, 38–40]. Like mRNAs, Pol II-derived pri-miRNAs are poly-adenylated at their 3′ end and bear 7-methyl-guanosine caps at their 5′ end [37]. The promoters of pri-miRNAs also contain CpG islands, TATA box sequences, initiation elements and certain histone modifications, indicating potential for regulation by transcription factors (TFs), enhancers, silencing elements and chromatin modifications [9, 35]. Therefore, many of the properties dictating the transcriptional regulation of miRNAs are the same as those regulating protein-coding genes. Following transcription, the stem-loop sequence of the pri-miRNA is recognized by a series of enzymes that orchestrate a tightly controlled maturation process.

## Pri-miRNA cleavage by the Microprocessor

In the canonical pathway, the pri-miRNA is cleaved in the nucleus by the RNase III enzyme Drosha into a ~60–70 nt pre-miRNA. Cleavage by Drosha requires the co-factor DGCR8 (DiGeorge critical region 8), also known as Pasha [41]. Together these two proteins comprise the minimum components of the Microprocessor complex (Fig. 1b). DGCR8 functions at least in part by binding to the junction between single-stranded and double-stranded regions of the pri-miRNA and directing Drosha to cleave approximately 11 bp downstream of this junction [42], generating products with 2 nt 3′ overhangs. It is thought that cleavage of the pri-miRNA by Drosha occurs co-transcriptionally along with splicing [43, 44], supported by the fact that Drosha co-localizes to sites of active transcription [45]. Processing of a pri-miRNA into a pre-miRNA can be regulated by a variety of protein co-factors that are either recruited to the Microprocessor through protein–protein interactions or through direct interactions with the pri-miRNAs.

### Regulation of pri-miRNA processing by proteins that interact with the Microprocessor

**Fig. 2:**
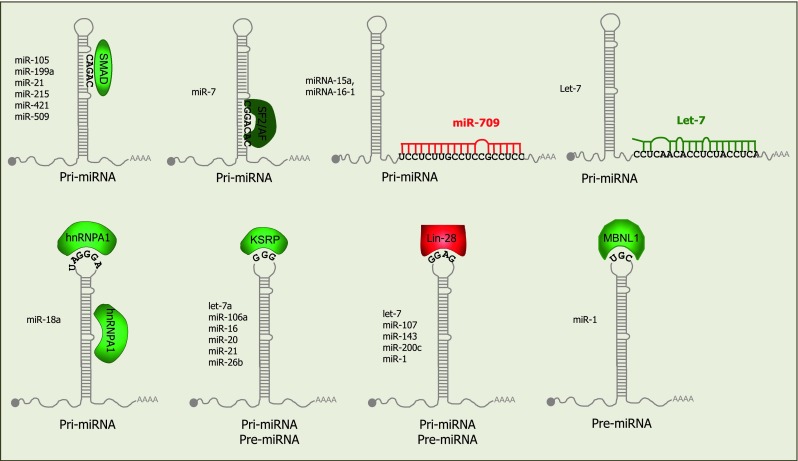
RNA motifs that mediate regulation of pri-miRNA or pre-miRNA processing. Proteins that positively (*green*) or negatively (*red*) regulate biogenesis associate with specific motifs in the stem-loop structures; depending on localization of the proteins, these either regulate the pri-miRNA or the pre-miRNA as listed below the hairpin; Lin28 and KSRP can regulate both forms. The identity of the miRNAs that contain the recognition motifs and have been validated to be regulated by each protein are listed to the left of the hairpin structure. Binding of miR-709 to pri-miR-15/16 inhibits its processing whereas binding of let-7 to pri-let-7 stimulates its processing

Many proteins have been identified that interact with Drosha, including the DEAD-box helicase proteins p68 (also known as DDX5) and p72 (DDX17) [41]. These helicases facilitate processing of nearly one-third of pri-miRNAs, according to studies with p68/p72 knock-out mice [46]. In some cases they do this by mediating interactions of TFs with the Microprocessor. A well-characterized example is the stimulation of maturation of specific pri-miRNAs by SMAD proteins, which are TFs induced upon stimulation with tumour growth factor β (TGF-β). The SMAD proteins associate with p68 to enhance processing through binding a consensus sequence in pri-miRNAs that strongly resembles the DNA SMAD-binding element (Fig. 2) [47–49]. Other TFs that regulate processing include the tumour suppressor p53, which promotes pri-miRNA processing via interaction with p68 [50] and ERα (estrogen receptor α), which inhibits the processing of specific pri-miRNAs via interactions with p68/p72 [51]. Another tumour suppressor, BRCA1 (breast cancer susceptibility gene 1), also associates with Drosha, p68, SMAD3 and p53 to accelerate processing of specific pri-miRNAs associated with cancer [52]. In contrast to the SMAD-regulated miRNAs, no consensus sequence has been identified within the miRNAs regulated by these TFs and the mechanisms underlying specificity in their regulatory functions are unknown. In addition to p68/p72, NF90 and NF45 (nuclear factor 90 and 45) also associate with the Microprocessor [41] and can inhibit processing of several miRNAs, including let-7 family members [53]. Other proteins that associate with Drosha and positively regulate processing include the multifunctional protein SNIP1 (SMAD nuclear interacting protein) [54] and ARS2 (arsenite-resistance protein 2) [55, 56]. However the precise mechanisms by which these multi-functional proteins influence biogenesis are unclear.

### Regulation of pri-miRNA processing by recognition of the stem-loop sequence or structure

Comparative analysis of pri-miRNA sequences suggests that 14 % of human pri-miRNAs have conserved nucleotides in their terminal loops, which may relate to interactions with regulatory proteins [57]. One of the first proteins identified to operate in this way was hnRNP-A1 (heterogeneous nuclear ribonucleoprotein A1), which binds to the terminal loop and stem of pri-miR-18a and facilitates processing by alteration of the stem structure [57, 58] (Figs. 1, 2). Interestingly, this protein can also interact with pri-let-7a, but in this case it negatively regulates processing [59]. The inhibitory effect appears to result from competition between hnRNP-A1 and KSRP (KH-type splicing regulatory protein), which both bind to the loop of pri-let-7a. KSRP positively regulates a subset of miRNAs and recognition has been proposed to derive from 2 or 3 sequential guanidines in the loop sequences [60] (Figs. 1b, 2). Interestingly, KSRP activity is modulated through its phosphorylation state in response to different stimuli and provides a link between PI3K/AKT signalling and miRNA processing [61, 62] (Figs. 1b, 2). Other RNA-binding proteins that interact with pri-miRNAs and promote their biogenesis include TDP-43 (TAR DNA-binding protein-43) [63] and the serine/arginine-rich SR protein SF2/ASF. The SF2/ASF protein binds to a motif in the stem of pri-miR-7 and has been proposed to alter the structure as observed for hnRNP-A1 [64]. Interestingly, miR-7 targets the 3′UTR of SF2/ASF, providing a negative feedback loop that may be important for controlling the steady-state expression level of this miRNA [64].

A key protein involved in regulating multiple aspects of miRNA biogenesis is Lin28 (abnormal cell lineage factor 28), which was originally discovered as a heterochronic gene regulating developmental timing in worms [65]. Lin28 can inhibit both pri-let-7 processing [66–68] and pre-let-7 processing [69–74] and recognition is mediated by the primary sequence and structure of the terminal loop (Fig. 2) [75]. Two Lin28 paralogs are present in mammals, Lin28A and Lin28B. Lin28A is predominantly cytoplasmic whereas Lin28B contains nuclear localisation signals and accumulates in the nucleolus. It has been proposed that Lin28B blocks let-7 processing by sequestering pri-let-7 miRNAs in the nucleoli away from the Microprocessor [68], suggesting a new mechanism by which other RNA-binding proteins might inhibit pri-miRNA biogenesis.

### Regulation of pri-miRNAs by other miRNAs

A recent study by Zisoulis and colleagues [76] demonstrates the pri-let-7 processing is also regulated by mature let-7, providing the first example of a direct auto-regulatory loop for let-7 biogenesis. In *C. elegans*, the ALG-1 (Argonaute-like protein-1) binds to a specific site at the 3′ end of the pri-let-7 and thereby promotes processing of the pri-miRNA. The interaction between ALG-1 and pri-let-7 is mediated by mature let-7 through a conserved site in the pri-miRNA transcript (Figs. 1b, 2). Immunoprecipitation of Ago proteins in human cells also suggests an interaction with pri-let-7, though it is not clear if this is mediated by a miRNA [76]. Interaction between a mature miRNA and a pri-miRNA can also have inhibitory effects on processing (Figs. 1b, 2). For example, miR-709 binds to a stretch of 19 nt in the sequence of pri-miR-15a/16-1, preventing pri-miRNA processing, leading to reduced levels of mature miR-15a/16-1 [77]. The factors underlying nuclear localisation of miR-709 remain unknown but this appears to be associated with apoptotic stimuli, and may be a dynamic mechanism for altering miR-15a/16 levels in response to external signals. Transfection of a miR-709 mimic into cells results in nuclear localisation of the synthetic RNA, indicating that the localisation signal is contained within the mature miRNA sequence. Nuclear localisation of miRNAs was first reported in a study showing that a hexanucleotide element within the mature miRNA sequence of miR-29b directs its nuclear transport [78]. However, this element is not present in miR-709 and the mechanism of nuclear transport is unknown. It appears that miR-709 and its binding site in pri-miR-15a/16 have co-evolved recently, as they are both only present in the mouse [77]. Further analyses are required to understand the breadth of regulation of pri-miRNAs by mature miRNAs and whether this relates to the nuclear localisation of Ago proteins that has been reported previously [79].

### The Drosha–DGCR8 regulatory loop and additional substrates of the Microprocessor

Regulatory feedback loops are thought to be a key feature of how miRNAs function in biological systems; for example, miRNAs that are induced by Toll-like receptor signalling target genes in this pathway, thereby dampening the inflammatory response [80]. The miRNA biogenesis machinery is also subject to regulation by feedback loops, as observed for the Drosha–DGCR8 complex [81–83]. DGCR8 stabilizes the Drosha protein in the Microprocessor complex and the Microprocessor complex in turn cleaves hairpin structures embedded in the 5′UTR of DGCR8 mRNA, leading to degradation of the DGCR8 transcript. This auto-regulatory loop is postulated to be critical to maintain the appropriate balance between the levels of the Drosha–DGCR8 complex and its substrates: when the Drosha–DGCR8 complex expression level is too low there is suboptimal miRNA processing; when the Drosha–DGCR8 complex expression level is too high, cleavage of non-miRNA substrates such as mRNAs may occur. Barad et al. [84] propose that efficient miRNA processing and minimal off-target cleavage is obtained only for a narrow range of Microprocessor concentration values. These studies also suggest that, apart from miRNA processing, the Microprocessor might play roles in mRNA stability control [83]. Consistent with this, HITS-CLIP analysis identified hundreds of mRNAs bound to DGCR8, including DGCR8 mRNA [85]. This study further demonstrated that cleavage within exonic cassettes can influence ratios of alternative spliced isoforms, suggesting complex roles of the Microprocessor in various modes of gene regulation. A viral mRNA was also shown to be regulated by Drosha in Kaposi's sarcoma-associated herpesvirus (KSHV) infection : the KapB (kaposin B) mRNA includes two pre-miRNAs in its 3′UTR and excision of these by Drosha alters the stability of the mRNA, thereby reducing KapB protein expression [86]. This mode of regulating viral gene expression during lytic or latent infection could represent an alternative function of viral miRNAs, where their processing serves a purpose, rather than (or in addition to) their activities in gene silencing.

## Regulation of pre-miRNA export

Once produced, the pre-miRNA is translocated to the cytoplasm through the nuclear pore complex by Exportin-5, which requires the co-factor RanGTP (Fig. 1) [24, 87, 88]. Structural analyses suggest that the length of the double-stranded stem and presence of 3′ overhangs are important for Exportin-5 recognition [1, 89]. Interestingly, Exportin-5 interacts with the RNA-binding protein NF90, also known as ILF-3 (interleukin enhancer-binding factor 3) [90], which is found in the Microprocessor complex [41]. It is possible that there is coordination between pri-miRNA cleavage and export but this has not been examined. Exportin-5 also shuttles tRNAs and other abundant RNAs to the cytoplasm and several studies suggest that export of pre-miRNAs can be regulated by these RNAs through competition. For example, Adenovirus produces a ~160 nt hairpin RNA (VA1 in Fig. 1c) that binds to Exportin-5 and inhibits nuclear export of pre-miRNAs [91]. Over-expression of short hairpin RNAs (shRNAs) in animals can also be toxic due to saturation of Exportin-5 and subsequent inhibition of pre-miRNA export [92]. Interestingly, Exportin-5 was also reported to interact with Dicer mRNA and high levels of pre-miRNAs or other Exportin-5 substrates can lead to accumulation of Dicer mRNA in the nucleus, providing another feedback loop for regulating the miRNA biogenesis factors [93]. The insect virus *Bombyx mori* nucleopolyhedrosis virus (BmNPV) negatively regulates nucleocytoplasmic transport of miRNAs by encoding a viral miRNA that targets RanGTP [94], although the functional relevance of this is not yet known.

## Dicer processing of pre-miRNAs

Once in the cytoplasm, the pre-miRNA hairpin associates with the RNase III-like enzyme Dicer that, in association with dsRNA binding domain (dsRBD) proteins, cleaves it into a double stranded miRNA duplex comprised of the mature miRNA and the miRNA* (or passenger strand) [25, 28, 95]. In flies, the dsRBD required for Dicer activity is Loquacious [96–98], whereas the proteins in mammals are TRBP (TAR RNA Binding Protein) and PACT (protein activator of PKR) [99–101]. In general, the thermodynamic asymmetry of the miRNA duplex determines which strand is incorporated in RISC: the miRNA strand whose 5′ end is less stably base-paired is more frequently retained [102, 103].

### Regulation of pre-miRNA processing: proteins and RNA motifs involved

Dicer-mediated processing of pre-miRNAs is subject to regulation by co-factors that interact with Dicer and RNA-binding proteins that recognize RNA elements within the pre-miRNAs. The Dicer protein alone can catalyse the cleavage of pre-miRNA, however, the specificity of cleavage is enhanced by TRBP and PACT [104]. Binding of TRBP and PACT also stabilizes Dicer and knockdown of TRBP and PACT reduces mature miRNA levels [99, 101]. TRBP also provides a link between MAPK (mitogen-activated protein kinase) signalling and miRNA processing since it is phosphorylated by Erk (extracellular signal regulated protein) [105]. The phosphorylated form of TRBP is more stable and leads to increased levels of many growth-promoting miRNAs in HEK293 cells and also causes a decrease in let-7 members. The mechanism for differential effects of phosphorylated TRBP on individual miRNAs is not yet clear [105].

The best-studied regulator of pre-miRNA processing by Dicer is Lin28 (Fig. 1d). Lin28A, the cytoplasmic isoform, binds a tetra-nucleotide sequence motif (GGAG) in the terminal loop of let-7 precursors and recruits TUT4 (terminal uridylyltransferase-4, also known as ZCCHC11), which adds an oligo U-tail to pre-let-7. This U tail blocks Dicer processing and mediates decay of pre-let-7, presumably through recruitment of 3′ to 5′ exonucleases [73, 106]. Lin-28A-dependent uridylation has also been observed for several other pre-miRNAs that contain the GGAG motif in their terminal loops, including miR-107, miR-143 and miR-200c [106, 107]. Kim and colleagues have recently shown that TUT4, as well as TUT2 and TUT7, can also add a single uridine to the 3′ end of a specific set of pre-miRNAs (termed “group 2” pre-miRNAs), which is independent of Lin28A. Up to 30 % of pre-let-7 family members have an untemplated uridine at the 3′ end in cells not expressing Lin28A [107, 108]. The pre-miRNAs that are modified lack a classical 2 nt 3′ end overhang, such that monouridylation results in the 2 nt overhang and thereby improves processing by Dicer [108].

Like Lin28, KSRP and TDP-43 are also involved in both pri- and pre-miRNA processing but they serve to promote, rather than inhibit, processing (Fig. 1b, d) [60, 63]. These findings suggest that the terminal loop is an important platform for both “activators” (for example, hnRNP A1, KSRP and TDP-43) and “repressors” (for example, Lin28) to modulate miRNA levels and thereby gene regulation, reviewed in [109]. There also appears to be some interplay between the activators and repressors. For example, the RNA binding protein MBNL1 (muscleblind-like splicing regulatory protein 1) binds to pre-miR-1 through recognition of a UGC motif that overlaps with a binding site for Lin28 (Fig. 2), such that MBNL1 binding blocks Lin28-mediated oligouridylation and subsequent degradation of pre-miRNA-1 [110]. Similar competition is seen with the mammalian immune regulator MCPIP1 (monocyte chemoattractant protein induced protein-1) and Lin-28: MCPIP1 is a ribonuclease that inhibits miRNA biogenesis by competing with Dicer for the cleavage of the terminal loop of pre-miRNAs. Addition of Lin28 abolishes MCPIP1-mediated cleavage in vitro, presumably through competition for binding to the terminal loop [111]. Other negative regulators of processing might also stabilize pre-miRNAs against degradation, but it is not clear if this is one of their functions in vivo. Recently Kouzarides’s group showed that Dicer processing can also be regulated by methylation of the 5′ end of the pre-miRNA by the human RNA-methyltransferase, BCDIN3D [112]. BCDIN3D adds two methyl groups to the 5′ phosphate of pre-miR-145 in vitro and in vivo; since Dicer specifically recognizes the 5′ monophosphate [113], this modification inhibits processing (Fig. 1d). A noncoding RNA in *C. elegans* was also shown to inhibit pre-miRNA processing: the ~800 nt noncoding RNA, rncs-1 (RNA noncoding, starvation up-regulated), competes with endogenous dsRNAs for binding to Dicer or accessory dsRBD proteins [114] (Fig. 1d). The VA RNAs in Adenovirus have also been shown to operate as competitive inhibitors for Dicer processing of pre-miRNA [91, 115], in addition to their inhibitory effects on Exportin 5.

Other viruses also inhibit this step in miRNA biogenesis. For example, Vaccinia Virus (VACV) infection leads to a drastic reduction in Dicer protein expression and a concomitant defect in pre-miRNA processing. The mechanism by which the virus abrogates Dicer expression remains unclear [116]. The human herpesvirus Epstein–Barr virus (EBV) influences Dicer processing through a more subtle mechanism: the viral-encoded miRNA miR-BART6-5p targets human Dicer mRNA [117]; it is expected that this could form a feedback loop to regulate the level of viral miRNAs. The host-encoded let-7 also regulates Dicer levels through target sites in the coding sequence, suggesting that feedback loops for controlling miRNA biogenesis may be inherent to miRNA homeostasis [118], which viruses can exploit.

## Regulation of miRNA expression by Argonaute proteins

MiRNAs function in partnership with Ago proteins, and a number of studies suggest that expression levels of miRNAs are tied to the expression levels of Agos. For example, ectopically expressed Ago proteins (Ago1–4) enhance expression of miRNAs under conditions where the miRNAs saturate the endogenous machinery [119], and endogenous miRNAs are reduced in mouse embryonic fibroblasts from Ago2-knockout mice [120]. Ago proteins are also subject to various levels of transcriptional and post-transcriptional regulation that might therefore influence miRNA expression. For example, the expression level of the Ago2 protein is specifically up-regulated in breast cancer cells lacking ERα, which is dependent on the EGFR/MAPK signalling pathway and leads to enhanced miRNA activity [121]. Ago2 can also be phosphorylated within the RNA binding pocket, which inhibits small RNA binding and is expected to thereby influence miRNA stability [122]. In addition to its role in miRNA stabilization, Ago2 has also been shown to catalyse an alternative pre-miRNA processing event [120]. Cleavage occurs within the 3′ arm of a pre-miRNA such that only the small RNA generated from the 5′ arm can be functional. The relevance of this alternative processing pathway remains elusive, but it may play a role in passenger strand dissociation for hairpins with a high degree of complementarity, where this might otherwise be inefficient [120].

## Non-canonical pathways of biogenesis: breaking the rules

**Fig. 3:**
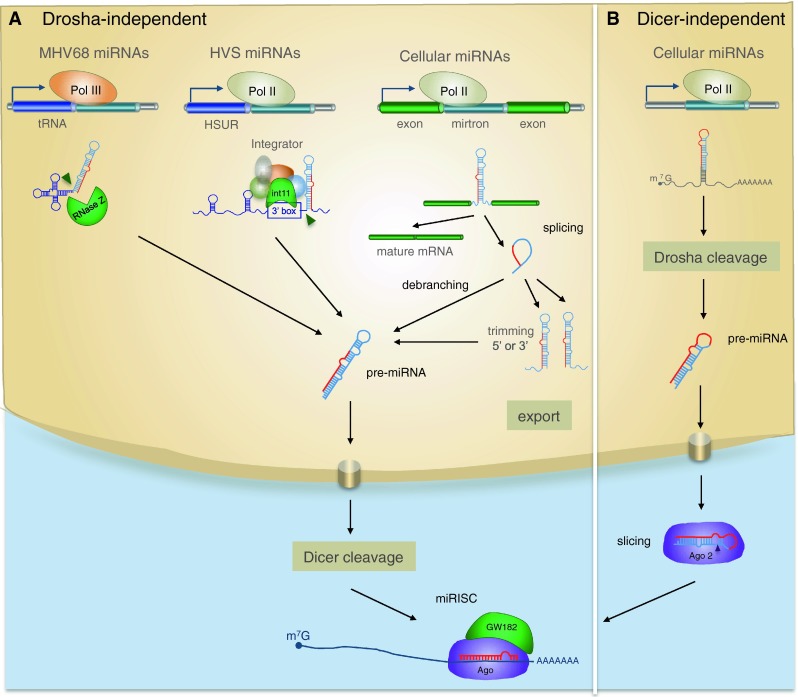
Alternative miRNA biogenesis pathways in animals and viruses. **a** Drosha-independent biogenesis. Pre-miRNAs are co-transcribed with tRNAs in Pol III transcripts in MHV68 and bypass processing by Drosha. Pre-miRNA like miRNAs in HVS are derived from the same Pol II transcripts as HSURs and require the Integrator for generation of their 5′ ends. Cellular miRNAs termed mirtrons also do not require Drosha: they are Pol II transcripts that are excised by splicing and linearized by lariat debranching; tailed mirtrons require further 5′ or 3′ trimming by nucleases and then they are directly processed by Dicer. **b** Dicer-independent biogenesis. The highly conserved miRNA, miR-451 is produced in a dicer-independent mechanism involving cleavage by Ago. The mature miRNA (*red*) derives from the stem as well as loop sequence of the pre-miRNA

In addition to the canonical biogenesis pathway, some miRNAs are processed by Drosha-independent and Dicer-independent pathways (Fig. 3) [123]. Studies of viral-encoded miRNAs in particular illuminate a range of non-canonical possibilities. For example, murine γ-herpesvirus 68 (MHV68) expresses its miRNAs in the same Pol III primary transcripts as the viral-encoded tRNAs [39, 40]. The pre-miRNAs are generated following cleavage by RNase Z and are subsequently processed by Dicer, thus bypassing the Microprocessor complex [124]. The retrovirus BLV also encodes Pol III-dependent pre-miRNA-like species that bypass Drosha cleavage and are subsequently processed by Dicer. Importantly, this mechanism provides a route for viral miRNA biogenesis that does not result in cleavage of the retroviral genomic RNA [18]. A miRNA-like species was also recently reported in West Nile virus (a cytoplasmic RNA virus) [16] and several reports have shown that artificial miRNAs engineered into RNA viruses are processed to a detectable level [125–127]. However, the mechanism(s) for biogenesis of these viral RNAs are not reported. Another alternative processing pathway has been described for miRNAs encoded by Herpesvirus Saimiri (HVS). These miRNAs are derived from the same Pol II transcripts that encode another class of viral noncoding RNA, HSURs (H. saimiri U-rich RNAs), which resemble small nuclear RNAs (snRNAs). The pre-miRNAs are located directly downstream of the 3′ end processing signals of HSURs and processing of the viral miRNAs does not require the Microprocessor [128]. Rather, the 5′ ends of the viral pre-miRNAs are produced by the Integrator, a nuclear complex of 12 proteins that associates with Pol II and is required for HSUR biogenesis. As in the canonical miRNA biogenesis pathway, HVS pre-miRNAs require Exportin-5 for transit to the cytoplasm, where they are processed by Dicer. An Integrator-dependent mechanism has not been reported for biogenesis of endogenous miRNAs. However, a range of reports suggest other mechanisms by which RNAs can be processed into miRNA-like species without a requirement for Drosha. For example, some miRNAs are derived from “mirtrons”, which are generated by splicing and debranching of short hairpin introns (Fig. 3) [129, 130]. The 5′ and 3′ ends are defined by donor and acceptor splice sites, but in some cases include additional unstructured tails [131, 132]. The biogenesis of 3′-tailed mirtrons in Drosophila was recently reported to utilize the RNA exosome, the major 3′–5′ exoribonuclease in eukaryotes [133]. Indeed, there is increasing overlap in the factors involved in miRNAs biogenesis and other RNA processing pathways. The list of RNAs that feed into the miRNA biogenesis pathway is also increasing: snoRNAs (small nucleolar RNAs), tRNAs and endogenous shRNAs can be processed by Dicer into small RNA fragments that then mediate gene silencing [131, 134–136].

Dicer is generally considered essential for the biogenesis of miRNAs, but at least one highly conserved miRNA, miR-451, is produced by a Dicer-independent mechanism in human, mouse and zebrafish [137–139]. The mature miRNA maps to the stem as well as loop sequence of the pre-miRNA and directly binds to Ago proteins (Fig. 3b). Ago1 and Ago3 can actively load pre-mir-451 but only Ago2 can process the miRNA since this requires the endonuclease activity [140]. To date, no other Dicer-independent miRNAs have been identified and the specific features that dictate routing to Dicer versus Ago are under investigation [140]. A recent report showed that pre-miRNAs could be designed to be processed by Ago2 as well as Integrator, eliminating the need for either Drosha or Dicer and opening up the possibility that such pathways could exist naturally [141].

## Regulation of miRNA biogenesis by single nucleotide polymorphisms and RNA editing

Natural sequence variations in pri-miRNAs, pre-miRNAs or mature miRNAs can influence their processing, stability and target selection. These sequence variations originate from changes in the DNA-coding sequence or from post-transcriptional modifications to the RNA [142–147]. In humans, differences in processing by Drosha were observed for alleles of miR-125a, miR-126, miR-146a, miR-502, miR-510, miR-890, and miR-892b [143–145, 147], while alteration of processing by Dicer was postulated for SNPs (single nucleotide polymorphisms) in miR-196a [146]. A natural variant of miR-934 was found to contain a mutation in the first nucleotide of the pre-miRNA, which affects strand selection for incorporation into RISC [145].

MiRNAs can also be post-transcriptionally modified by the ADAR family members (adenosine deaminase acting on RNA proteins) which convert adenosines to inosine, reviewed in [148]. The hairpin structures of pre-miRNAs are favourable substrates for ADARs [149], which recognize dsRNA. Blow et al. [150] sequenced 99 miRNAs from 10 human tissues and identified 6 % of pri-miRNA transcripts with A to I conversions in at least one of the analysed tissues. Another survey reported that 16 % of pri-miRNAs are edited in the brain, where there is generally a higher frequency of RNA editing [151]. Editing can affect pri-miRNA and pre-miRNA processing and can also alter the target repertoire of the miRNA when editing occurs in the mature sequence [152–155]. For example, editing of pri-miR-142 substantially reduces processing by Drosha and leads to cleavage by Tudor-SN (Tudor staphylococcal nuclease), a component of RISC with ribonuclease activity specific for inosine-containing dsRNAs [154, 155]. In contrast, editing of pri-miR-151 by ADAR1 does not affect pri-miRNA processing but interferes with pre-miRNA cleavage by Dicer, as seen by accumulation of edited pre-miR-151 (Fig. 1d) [153]. The A to I conversion within the mature miRNA can retarget the miRNA to a new set of mRNAs since inosine base pairs with cytosine rather than uridine. For example, editing of sites within the miR-376 seed alters its target repertoire both in vitro and in vivo [152]. Interestingly, Heale et al. reported that ADAR enzymes can also influence miRNA processing independently of their catalytic activity, suggesting that in some cases binding of the ADAR proteins alone might be sufficient to interfere with miRNA processing [156].

Some viral miRNAs have also been found to be edited, for example KSHV miR-K12-10 [40], Marek’s disease virus miR-M7 [157] and EBV miR-BART6 [117]. To date the functional relevance of this editing has only been suggested for the latter. In HEK-293 cells, editing of EBV miR-BART6-3p decreases the efficiency with which the miRNA encoded on the opposite strand, miR-BART6-5p, is loaded into RISC. Strikingly, miR-BART6-5p targets human Dicer via 4 binding sites in its 3′UTR. Therefore, editing of miR-BART6-3p relieves Dicer from posttranscriptional gene silencing. Dicer levels affect the expression levels of multiple genes that regulate the infectious and lytic states of EBV and it is postulated that editing of miR-BART6-3p could be an indirect way to modulate miRNA biogenesis and thereby the viral life cycle [117].

## Regulation of miRNA stability

Once a mature miRNA is incorporated into RISC it is generally considered to be extraordinary stable [158, 159]. Indeed, upon inactivation of miRNA transcription or processing the majority of mature miRNAs in human and rodent cell lines have half-lives in the range of many hours to days [160, 161]. However, recent reports from various model systems have demonstrated differences in the stabilities of individual miRNAs, suggesting that regulated degradation of specific miRNAs is a physiologically relevant way to modulate their expression, reviewed further in [162]. In particular, active miRNA decay seems to play a prominent role in neurons. In mouse retinal cells the sensory neuron-specific miR-183/96/182 cluster and miR-204 and miR-211 are differentially expressed in response to light. The mature miRNAs are rapidly down regulated upon dark-adaptation due to active degradation by a yet unidentified enzyme [163]. Several other brain-enriched miRNAs have short half lives both in primary human neuronal cell culture and post mortem brain tissue [164]. The fast turnover is recapitulated in primary neurons outside the retina as well as in neurons derived from mouse embryonic stem cells. Strikingly, blocking of action potentials by inhibition of sodium channels prevented the degradation of selected miRNAs, indicating that activation of neurons is required for the regulated decay of some neuronal miRNAs [163]. In line with this observation, a small RNA deep sequencing approach identified several brain-enriched miRNAs that also were rapidly down regulated upon transient exposure to the neurotransmitter serotonin in the marine snail *Aplysia* [165]. Active miRNA decay represents an elegant way to re-activate neuronal transcripts, which might be important for a rapid response to various external stimuli [166–169]. Regulated miRNA turnover also occurs during viral infection (described below), although to date the mechanisms of miRNA turnover in neurons or during infection in mammals remain unknown. However, studies from other model systems have identified molecular determinants of regulated miRNA decay and here we will summarize the current knowledge on these determinants and their modes of action.

### Modifications to the 3′ end of miRNAs

Chemical modifications of mature miRNAs play a crucial role in regulating their stabilities. The first appreciation for miRNA stability factors came from studies in plants, where the methyltransferase HEN1 (Hua enhancer 1) methylates the 2′ hydroxyl group of the 3′ terminal nucleotide of a miRNA [170–172]. Methylation of plant miRNAs protects their 3′ ends from terminal uridylation by the nucleotidyl transferase HESO1 (HEN1 suppressor 1), which triggers their degradation [173–175]. Uridylation at the 3′ ends of RNAs is also associated with reduced stability of piRNAs, siRNA and mRNAs [176–178]. Similarly, a nucleotidyl transferase in the unicellular alga *Chlamydomonas reinhardtii*, MUT68, uridylates small RNAs leading to their degradation by the peripheral exosomal subunit RRP6 (ribosomal binding protein 6) [179].

Animal miRNAs generally lack a protective 2′-*O* methyl group at their 3′ terminus and display template-independent nucleotide addition, mostly adenylation or uridylation that may regulate miRNA stability [180–182]. Several enzymes, including MTPAP, PAPD4/GLD2, PAPD5, ZCCHC6, TUT4/ZCCHC11, and PAPD2/TUT1 display terminal nucleotidyl transferase activity and knockdown experiments indicate that these proteins are responsible for miRNA 3′ end variation to various extents [183, 184]. However, functional implications have thus far been described for only a few of these enzymes. For example, TUT4, the nucleotidyl transferase implicated in the degradation of histone mRNA and several pre-miRNAs [73, 110, 178], regulates cytokine levels by uridylation of mature miR-26 family members [185]. In the human A549 cell line, miR-26b targets the IL6 (interleukin 6) transcript but terminal uridylation of this miRNA interferes with its function. Knockdown of TUT4 results in reduced miR-26a uridylation along with decreased expression of a reporter containing the IL6 3′UTR. Conversely, overexpression of TUT4 leads to enhanced levels of the same reporter, indicating that uridylated miR-26a is less effective in targeting IL6. Notably, knockdown of TUT4 does not increase miR-26 expression levels, indicating that uridylation of the miRNA affects its activity without affecting its expression [185].

Adenylation at the 3′ ends of miRNAs is associated with both enhanced and decreased miRNA stability [186–189]. For example, the most highly expressed miRNA in the liver, miR-122, is monoadenylated by the cytoplasmic poly(A) polymerase GLD2 (germline development defective-2). In GLD2 knockout mice, miR-122 is selectively destabilized whereas the levels of 10 other miRNAs remain unchanged. The stability of the miR-122 precursors is not affected by GLD2 knockout, suggesting a role for adenylation in modulating stability of the mature form [186].

Recently it was demonstrated that VACV induces polyadenylation of endogenous miRNAs during infection. The viral poly(A) polymerase is responsible for the non-templated adenylation that results in a ~30-fold reduction of endogenous miRNA levels in infected mouse embryonic fibroblasts; other small RNAs such as tRNAs and snRNAs remain largely unaffected by VACV infection. It was suggested that viral poly(A) polymerase operates only on Ago-bound small RNAs, but the mechanism is unknown. Whereas polyadenylation of miRNAs is mediated by a viral gene product, the actual degrading activity is postulated to stem from a yet undefined cellular protein [189]. It is not clear if and how the modification of miRNAs by VACV is linked to the reduction in Dicer expression that was described previously [116]; it may be that this virus uses two different mechanisms to shut-off cellular miRNA expression. Poxviruses infect a wide range of vertebrate and invertebrate hosts. Infection of *Drosophila* cells with VACV leads to global reduction in miRNA expression whereas the levels of endogenous siRNAs are unaffected. Like plant miRNAs, insect siRNAs are methylated, which protects them from polyadenylation by the virus. Indeed 3′ methylation of a transfected miRNA prevents it from being polyadenylated and degraded during infection [189]. The advent of deep sequencing technology has enabled a much greater appreciation for the extent of heterogeneity and modifications at the 3′ ends of miRNAs [182, 183, 190]. In the coming years it will be important to further characterise the enzymes that write and read these modifications and to understand their impact on miRNA stability and function.

### Sequence motifs regulating miRNA stability

Several reports have demonstrated altered kinetics in the turnover of individual miRNAs under conditions where the expression levels of most miRNAs are unchanged [160, 161]. This suggests that *cis* acting elements in the mature miRNA sequence provide specificity to the miRNA degradation process. In a survey to characterise the role of miRNA turnover during the cell cycle, Rissland and colleagues [191] found that miR-503 and other members of the extended miR-16 family are constitutively unstable in NIH-3T3 cells. The high turnover rate allows dynamic transcriptional regulation of these miRNAs during the cell cycle. For example, miR-503 is rapidly down regulated upon cell cycle re-entry but accumulates during cell cycle arrest by serum starvation. Sequence elements within the seed and 3′ end of the miRNA appear to be required for the degradation. Similarly, miR-382 is selectively unstable in HEK293 cells and an element in the 3′ end of the miRNA is required for its enhanced turnover in vitro [160]. Optimal paradigms to study *cis* acting elements with a role in miRNA decay are miRNAs that are co-transcribed and highly similar on a sequence level, yet differ in their decay rates. The miR-29 family provides such an example: miR-29b is unstable in cycling cells and only accumulates during mitosis whereas miR-29a is stable throughout the cell cycle [78]. The miR-29a and miR-29b share the same seed sequence but are distinguished by a C to U substitution at position 10 and miR-29b contains a hexanucleotide motif (AGUGUU) at its 3′ end that is responsible for its nuclear localisation. However, the motif does not account for the accelerated miRNA decay. Instead, uridines at position 9–11 in miR-29b seem to enhance destabilisation and many, but not all, miRNAs that contain a uridine stretch at this position are reported to display faster turnover rates [192]. Therefore, additional factors must dictate the differential stability of miRNAs. Altogether these studies show that miRNAs, though limited in coding space, contain sequence elements outside the classical seed that may critically influence miRNA abundance and function. To date, no viral miRNAs have been reported to contain such motifs, but this could provide another strategy for viruses to diversify miRNA function and regulation during their life cycles. Identification of the *trans*-acting factors that recognise these motifs is important for further investigations in this area.

### *Trans*-acting factors regulating miRNA stability

The first report of enzymes that are capable of degrading single-stranded small RNAs came from a candidate gene approach in plants. In *Arabidopsis*, SDN1 (small RNA degrading nuclease 1) possesses 3′–5′ exonuclease activity on small RNAs including miRNAs. In a cell free assay system, SDN1 specifically degrades ssRNA but not dsRNA. The 2′ *O*-methylation present on the 3′ terminal nucleotide of plant miRNAs is protective against SDN1 activity [193]. The enzyme belongs to a family of exoribonucleases with partially overlapping functions in vivo that are responsible for miRNA turnover in plants. Interestingly, members of this protein family are conserved in all eukaryotes and it seems likely that animal homologues of SDNs have similar functions but these have not yet been reported [194]. The XRN family of enzymes play various roles in miRNA stability in different organisms: in *Arabidopsis*, XRN2 and XRN3 are involved in degrading the loop sequence of pre-miRNAs [195], in mammalian cells, XRN2 degrades the pri-miRNA following processing by Drosha [43, 196]. In *C. elegans,* XRN2 degrades mature miRNAs once released from the RISC complex and may also influence the rate at which they are released [197]. Interestingly, the presence of target RNA counteracts the decay of miRNAs by XRN2 both in vitro and in vivo [197, 198]. Whether this is due to direct competition between the target and XRN2 for miRNA binding or through another molecular mechanism is not yet known. The exoribonuclease XRN1 and the exosome core subunit Rrp42 (ribosomal RNA-processing protein-42) are proposed to be involved in turnover of miR-382 in HEK293 cells, as knock-down of these factors selectively increases miR-382 expression levels [160].

In a human melanoma cell line, ectopic expression of hPNPase^old-35^ (human polynucleotide phosphorylase protein) leads to the selective down regulation of several miRNAs (miR-221, miR-222 and miR-106b). Immunoprecipitation studies show that this 3′–5′ exoribonuclease directly associates with these miRNAs and causes their degradation in vitro. However, it remains unclear whether hPNPase^old-35^ is also able to actively dislodge them from the RISC complex. Interestingly, hPNPase^old-35^ is an interferon-stimulated gene and mediates IFN-β-induced down regulation of miR-221. One of the direct targets of miR-221 is the cell-cycle suppressor p27^kip1^. Consequently, both miR-221 overexpression and knockdown of hPNPase^old-35^ protect human melanoma cells from INF-β- induced growth arrest, indicating a pivotal role of controlled miRNA decay in tuning cell proliferation [199]. The 3′–5′ exoribonuclease Eri1 was recently implicated in regulating miRNA stability in mouse lymphocytes, based on the global increase in miRNA levels observed in NK and T cells from Eri1 knockout mice [200]. The regulation of miRNA levels by Eri1 appears to be required for NK-cell development and antiviral immunity, but its mechanism of action remains to be established.

Besides promoting miRNA degradation, RNA binding proteins can also enhance the stability of mature miRNAs. For example, Quaking, a member of the STAR (signal transduction and activation of RNA) family of RNA binding proteins, is up regulated in response to p53 signalling and stabilises mature miR-20a [201]. The identification of proteins that stabilise and de-stabilise mature miRNAs supports the idea that regulation of miRNA decay is important in controlling the miRNA repertoire of the cell. Yet, there are still major gaps in understanding how specificity in degradation or stabilization is mediated.

### Target mediated miRNA turnover

**Fig. 4:**
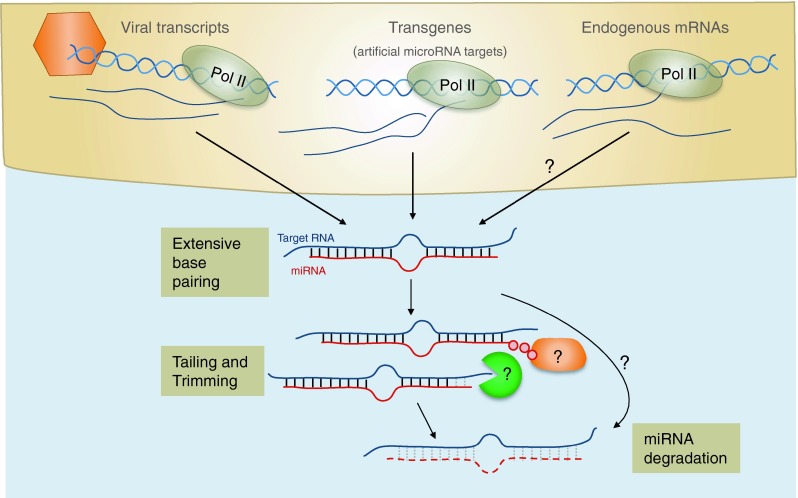
Target-mediated miRNA degradation. Different sources of target RNA can induce miRNA decay including two herpesviral transcripts (Herpesvirus saimiri HSUR1 and murine Cytomegalovirus m169) and transgenic expressed miRNA targets with extensive basepairing. Whether there are endogenous mRNAs that induce miRNA degradation remains to be investigated. Both in vertebrates and invertebrates target-mediated miRNA degradation has been associated with tailing and trimming of miRNAs. The relationship between tailing and trimming is still unclear, and the factors involved in mediating these effects and subsequent degradation remain to be determined

In contrast to target-mediated stabilization of miRNA in *C. elegans*, binding of miRNAs to RNAs can promote miRNA degradation in *Drosophila* and mammals. In flies, most miRNAs are incorporated in Ago1-containing RISC complexes whereas siRNAs, usually derived from dsRNA from viruses and transposons, are loaded into Ago2 [202] and are 3′ methylated by the Drosophila homolog of HEN-1 [203]. Intriguingly, binding of Ago1 associated miRNAs to target sites with extensive complementarity results in destabilization of the miRNAs [204]. Deep sequencing the small RNAs revealed that a large proportion of these miRNAs are either shortened or have non-templated nucleotide additions at their 3′ ends (mostly adenines and uridines). This mechanism of trimming and tailing, mediated by as yet unknown enzymes, seems to precede miRNA decay (Fig. 4). In contrast, miRNAs that associate with Ago2 and thus are methylated appear to be protected from degradation. In human cells, miRNAs are also subject to this target-directed destabilisation, as evidenced by trimming and tailing in Hela cells in vitro [204]. Baccarini and colleagues [205] examined in more detail the fate of a miRNA molecule after target recognition and demonstrated that miRNAs generally out-live their targets, whether the target is perfectly complementary or contains a central bulge. However, target recognition promotes post-transcriptional modification of miRNAs (mostly 3′ uridylation) which is postulated to induce their degradation, thereby limiting miRNA recycling. It is not yet known what features in the target RNA direct the posttranscriptional modification of a miRNA but this may involve extensive pairing as proposed in flies [204].

Two distinct mammalian herpesviruses, a gamma herpesvirus infecting new world primates and a beta herpesvirus infecting mice, exploit the mechanism of target-directed miRNA degradation (Fig. 4). Several HSURs are expressed in *Herpesvirus saimiri* (HVS)-transformed T-cells and one of these, HSUR1, contains an interaction site for the endogenous miRNA, miR-27 [206]. Cazalla et al. [206] showed that binding of HSUR1 to miR-27 accelerates its rate of turnover and replacing the miR-27 interaction site with a binding site for miR-20 re-targets HSUR1 to the other miRNA. Similarly, miR-27 is also rapidly down regulated in murine cytomegalovirus (MCMV) infection of several mouse cell lines as well as primary macrophages. Yet, the expression levels of miR-27 precursors remain stable, indicating that the mature form is subject to enhanced degradation, presumably by a viral inhibitor [207]. Indeed, the MCMV *m169* gene contains a binding site for miR-27 in its 3′ UTR and miR-27 levels are rescued if the m169 gene is knocked down or deleted from the virus [208, 209]. During lytic MCMV infection, *m169* is among the most highly transcribed genes [209] and it represents the most frequent non-miRNA segment sequenced in Ago2 immunoprecipitations [208]. Down regulation of miR-27 is linked to its 3′ end tailing and trimming, indicating that a similar mechanism as suggested in flies and human cells might underlie the degradation process [204, 209]. As reported for HSUR1, replacing the miR-27 binding site with an interaction site for an unrelated miRNA is able to redirect *m169* to target that specific miRNA [206, 208, 209]. The degradation of miR-27 by two distinct herpesviruses might suggest that this miRNA plays an important role in the viral life cycles. Indeed, miR-27 represses MCMV replication when over-expressed in cell culture experiments [207] and MCMV mutants incapable of down regulating miR-27 display attenuated viral growth in mice [209]. So far, however, it remains unclear which cellular miR-27 target(s) are responsible for modulating MCMV replication and whether it plays the same role in both MCMV and HVS infections. In summary, the pairing patterns of miRNAs with their targets as well as the relative amounts of each seem to be crucial factors that determine the extent of target-mediated miRNA decay [205]. A range of reports suggest that endogenous mRNAs, noncoding RNAs and pseudogenes also play a role in regulating miRNA activity and/or stability, reviewed in [210].

## Viral suppressors of RNA interference may modulate miRNA expression

In insects and plants, RNA silencing pathways mediate a potent antiviral response. For efficient replication, viruses that infect these hosts therefore rely on virus-encoded suppressors of RNAi (VSRs) [211]. Also in mammalian viruses, proteins with RNAi suppressive activity have been identified, although the importance of this suppressive activity in vivo remains to be established [212, 213]. In the following section we will discuss how the expression of these VSRs affects miRNA biosynthesis in insects and plants and we will further speculate about their possible influence on miRNA expression in mammals.

The RNA interference machinery in insects recognizes viral dsRNA in the cytoplasm and processes it into vsiRNAs (viral siRNAs) [211]. These vsiRNAs associate with Ago2-containing RISC complexes, which then act as antiviral effectors by cleaving viral RNA in the cytoplasm [214]. Whereas the production of siRNA and miRNA molecules in mammals largely rely on the same biogenesis factors, the miRNA and antiviral RNAi pathways in insects are governed by a distinct set of processing and effector complexes. Specifically, pre-miRNAs are processed by Dicer-1 to be loaded into Ago1-containing RISC complexes. In contrast, cytoplasmic long dsRNA is sensed and cleaved by Dicer-2 and the resulting 21 nt siRNAs are predominantly loaded into Ago2-containing RISC [202, 215, 216]. Insect VSRs interfere with the RNAi machinery at different stages of the pathway. Drosophila C virus 1A for example binds long dsRNA, thereby preventing its efficient processing into siRNA [214]. Flock house virus B2 binds both long dsRNA and siRNAs [217–220]. Cricket Paralysis virus 1A and Noravirus VP1 directly interact with the small RNA-loaded Ago2 effector complex and prevent its target RNA cleavage activity [221, 222] (and unpublished observations).

Although the siRNA and miRNA biogenesis machineries are distinct in insects and plants, many VSRs have dsRNA binding properties, and it might be expected that they could affect miRNA processing too. However, this does not seem to be the case in flies. VSR expression in transgenic *Drosophila* does not alter levels of mature miRNAs, nor does it affect the activities of miRNA reporters. Furthermore, in contrast to Ago1 loss-of-function mutants, transgenic animals expressing VSRs do not display developmental defects, suggesting that VSRs do not affect global miRNA biogenesis and function [214, 219, 221–223]. In contrast, transgenic expression of VSRs in plants leads to pleiotropic, developmental defects due to alterations in miRNA-mediated gene regulation [224–226]. This is likely based on the convergence of the plant siRNA and miRNA biogenesis pathways, which share several processing factors. For instance, both miRNAs and antiviral siRNAs can be loaded into Ago1 effector complexes in plants [227–229]. Yet, for many plant VSRs, it remains elusive how they manipulate the miRNA machinery in vivo. A number of VSRs have dsRNA binding activity in vitro, which has been hypothesised to explain their interference with miRNA biogenesis [230–235]. For instance, Tombusvirus P19 directly binds siRNA duplexes preventing their efficient loading into effector RISC complexes in vitro [224, 225, 230, 236–238]. In transgenic *Arabidopsis,* P19 also prevents miRNA loading into Ago1-containing RISC. However, this seems to be a rather exceptional property as three other VSRs tested, Turnip crinckle virus P38, Peanut Clump virus P15, and Turnip mosaic virus Hc-Pro, blocked siRNA loading into Ago1 but did not disturb its association with miRNAs [238].

A number of plant VSRs may act on the miRNA machinery in other ways than by small RNA sequestration. Turnip crinckle virus (TCV) P38 and Sweet potato mild mottle virus (SPMMV) P1 directly interact with the siRNA/miRNA effector Ago1 by mimicking the glycine/tryptophan (GW)/WG repeats normally found in host proteins that associate with Ago proteins [239, 240]. Indeed, host miRNA levels were reduced in TCV infections [240] and P1 expression suppresses silencing of a miRNA sensor [239]. However, in a study using transgenic *Arabidopsis*, P38 did not suppress accumulation of miRNAs in Ago1-containing RISC complexes [238], which might reflect the differences between the two model systems (TCV infection versus P38 transgenic plants). Beet western yellow virus P0 has been suggested to target Ago1 for degradation by acting as a F-box protein [241–244]. F-box proteins are components of E3 ubiquitin ligase complexes, which target proteins for ubiquitination and subsequent proteasomal degradation [245]. Interestingly, the VSR activity of P0 is insensitive to proteasome inhibition, indicating that P0 induces Ago1 degradation via a non-canonical pathway [241]. Besides suppression of dsRNA-induced RNAi, transgenic expression of P0 in *Arabidopsis* causes developmental defects reminiscent of miRNA pathway-defective plants. Indeed, six out of twelve analysed miRNA target genes have elevated expression levels suggesting that P0 also affects the miRNA pathway [242]. The indications that P38, P1 and P0 inhibit both (v)siRNA and miRNA biogenesis may reflect the convergence of these two pathways on Ago1 [227–229].

In mammalian cells, virus infection triggers a potent protein-based immune response and it remains unclear to what extent RNAi-based mechanisms contribute to antiviral immunity. Yet, three lines of evidence support the idea that vsiRNAs could contribute to antiviral immune defence in mammals. First, in a broad small RNA deep-sequencing survey of six different RNA virus infecting multiple hosts, virus-derived small RNAs were discovered in 4 positive (+) strand RNA viruses and 1 negative (−) strand RNA virus [246]. However, the origin, Dicer-dependence, and functional importance of these small RNAs remains to be established. Second, siRNAs engineered to target viruses restrict virus growth in several mammalian model systems [247, 248]. This suggests that the RNAi pathway could have intrinsic antiviral activity, provided that vsiRNAs are naturally generated at sufficient levels. Third, several viruses were suggested to encode proteins that suppress RNAi in mammalian cells, including Influenza virus NS1, Vaccinia virus E3L, Nodamura virus B2, La Crosse virus NSs, HIV Tat and Ebola virus VP30, VP35 and VP40 [216, 249–253]. Many of these VSRs, including NS1, E3, VP30 and VP35, have dsRNA binding activity. Influenza NS1 protein has been demonstrated to function as VSR only in heterologous plant and *Drosophila* cell systems [216, 254, 255]. In mammalian cells this protein fails to suppress RNAi induced by exogenous shRNA or siRNAs [256]. The VSR activity of Nodamura virus B2 has also been attributed to its RNA binding properties. The B2 binds both siRNAs and shRNAs and interferes with Dicer processing in mammalian cells in vitro [249]. Since pre-miRNAs are structurally similar to shRNAs, it is expected that this VSR could bind pre-miRNAs and thereby hinder their processing. Indeed, human cells stably expressing NoV B2 display elevated levels of pre-let-7d, suggesting that efficient Dicer processing of this pre-miRNA is inhibited [249]. However, this effect was not observed for two other endogenous miRNAs and the mechanism has not been examined further [249]. Nonetheless, these results demonstrate that viral RNA binding proteins have the potential to interfere with miRNA biogenesis through RNA–protein interactions.

In contrast to RNA binding, VSRs may also function through direct interaction with protein components of the mammalian RNAi machinery. Ebola virus VP30 and VP35 can directly interact with Dicer or with Dicer-associated factors TRBP and PACT, and thereby inhibit the production of functional siRNAs [252, 253]. Unlike the small RNA biogenesis machinery in insects, mammalian cells only express one Dicer that is responsible for both the production of siRNAs and miRNAs [5]. Inhibition of Dicer processing by VP30 and VP35 is, therefore, expected to interfere with pre-miRNA processing but this requires further experimental validation. Similarly, the HIV Tat protein has been suggested to interfere with Dicer processing of shRNAs in vitro [251]. Tat associates with Dicer in an RNA-dependent manner but the molecular identity of the required RNA is still unknown [257]. Furthermore, it remains elusive if the Tat-Dicer interaction is necessary for the VSR activity of Tat. A retrovirus, Primate foamy virus (PFV) type 1 encodes the Tas protein, which has been suggested to be a non-specific suppressor of miRNA-mediated silencing with an as yet unknown mode of action [258]. Interestingly, PFV is efficiently targeted by the host miR-32 and inhibiting this cellular miRNAs with locked nucleic acid miRNA antagonists enhances PFV replication. Blocking the miRNA-virus interaction may thus represent a major function of Tas VSR activity. However, the antiviral activity of miR-32 remains an item of debate [259], as does the functional importance of retroviral VSRs. For example, Qian et al. [260] suggest that HIV Tat protein suppresses RNAi by inhibiting a step downstream of siRNA processing. In another study, overexpression of both HIV tat and PFV Tas failed to suppress shRNA-induced RNAi in human cells [261].

To conclude, a number of mammalian VSRs have the potential to actively manipulate host miRNA biogenesis either through interactions with RNA or protein components of the small RNA processing machinery. Yet, for most candidate VSRs, firm support for a global change of miRNA levels or activity in the context of an authentic infection is lacking. Making use of high throughput sequencing and screening approaches it will be possible to assess to what extent VSRs contribute to changes in miRNA expression or activity in infected mammalian cells.

## Conclusions

Since their initial discovery nearly 20 years ago miRNAs have been shown to play fundamental roles in virtually all cell-biological processes. Therefore it is not surprising that their expression is tightly regulated in a spatio-temporal fashion. There are many mechanisms by which miRNAs can be produced and subsequently regulated in mammalian cells. Studies of viral systems have revealed diversity in the origin of miRNAs, the factors required for their synthesis, and the factors that can control their turnover. In some cases, viruses influence global expression levels of miRNAs, in line with their mode of action in targeting RNAi pathways in plants and insects. However, as reviewed here, miRNAs play diverse functional roles in a cell and there are numerous mechanisms for regulating specific subsets of miRNAs, or individual miRNAs, rather than the global machinery. It appears that some viruses such as HVS and MCMV have tapped into these modes of regulation, most likely in order to precisely control specific pathways in the host cell. With the advancement of RNA–protein mapping techniques and sequencing technologies, it is likely that many more viral-host interactions targeting miRNA regulation will emerge.
